# NMR ^1^H,^13^C, ^15^N backbone and ^13^C side chain resonance assignment of the G12C mutant of human K-Ras bound to GDP

**DOI:** 10.1007/s12104-018-9821-8

**Published:** 2018-05-02

**Authors:** Alok K. Sharma, Seung-Joo Lee, Alan C. Rigby, Sharon A. Townson

**Affiliations:** Warp Drive Bio, Inc., 400 Technology Square, Cambridge, MA 02139 USA

**Keywords:** Ras family, K-Ras, Cell proliferation, G12C, Cancer, HSQC, NMR

## Abstract

K-Ras is a key driver of oncogenesis, accounting for approximately 80% of Ras-driven human cancers. The small GTPase cycles between an inactive, GDP-bound and an active, GTP-bound state, regulated by guanine nucleotide exchange factors and GTPase activating proteins, respectively. Activated K-Ras regulates cell proliferation, differentiation and survival by signaling through several effector pathways, including Raf-MAPK. Oncogenic mutations that impair the GTPase activity of K-Ras result in a hyperactivated state, leading to uncontrolled cellular proliferation and tumorogenesis. A cysteine mutation at glycine 12 is commonly found in K-Ras associated cancers, and has become a recent focus for therapeutic intervention. We report here ^1^H^N, 15^N, and ^13^C resonance assignments for the 19.3 kDa (aa 1–169) human K-Ras protein harboring an oncogenic G12C mutation in the GDP-bound form (K-RAS^G12C-GDP^), using heteronuclear, multidimensional NMR spectroscopy. Backbone ^1^H–^15^N correlations have been assigned for all non-proline residues, except for the first methionine residue.

## Biological context

Ras GTPases play a critical role in regulating pathways involved in cell growth, proliferation, differentiation and apoptosis (Hunter et al. 2014; Ostrem et al. 2013). The activity of Ras is modulated by inter-conversion between an inactive, GDP-bound state and an active, GTP-bound state, which is tightly regulated by numerous proteins including guanine nucleotide exchange factors (GEFs) and GTPase activating proteins (GAPs) (Pylayeva-Gupta et al. 2011). Approximately 20–30% of human tumors are attributed to somatic mutations in Ras proteins (Downward 2003). Oncogenic mutations at positions 12, 13, or 61 attenuate the GTPase activity of Ras resulting in uncontrolled functional hyperactivation. Elevated levels of active Ras in turn contribute to several aspects of malignant phenotypes, leading to cancer. Amongst the three Ras isoforms (K-Ras, H-Ras, and N-Ras), oncogenic mutations are more frequently observed in K-Ras and are estimated to account for over 80% of all Ras-driven cancers (Prior et al. 2012). In particular, K-Ras harboring a cysteine mutation at position 12 is commonly found in K-Ras associated cancers, including lung, pancreatic, and colorectal adenocarcinomas (Stephen et al. 2014; Prior et al. 2012), and has become a target for therapeutic intervention using cysteine-reactive covalent small molecule inhibitors (Janes et al. 2018; Prior et al. 2012; Ostrem et al. 2013).

K-Ras contains a guanosine nucleotide-binding domain (G domain of ~ 20 kDa) at the N-terminus and a short hypervariable region at the C-terminus. Several regions within the G domain are particularly important for nucleotide exchange and downstream signalling, including the P-loop (residues 10–17) that binds nucleotide phosphate, as well as the Switch I (25–40) and Switch II (57–75) regions that interact directly with GEFs (Boriack-Sjodin et al. 1998) and effector proteins, such as Raf and RalGDS (Downward 2003). Analysis of the protein NMR assignments of GTP- and GDP-bound forms of wild-type (WT) K-Ras reveal major differences in residues in the G domain, localized within the P-loop, Switch I and Switch II regions (Vo et al. 2013; Boriack-Sjodin et al. 1998; Downward 2003). For GTP-bound Ras, local polysterism and conformational exchange render residues in the aforementioned regions undetectable in the fingerprint region of the 2D ^1^H–^15^N HSQC spectrum under physiological conditions (Ito et al. 1997; O’Connor and Kovrigin 2012). In contrast, residues in the entire G domain are observed in the 2D ^1^H–^15^N HSQC spectrum of GDP-bound Ras (Muto et al. 1993; Gossert et al. 2011; Vo et al. 2013; Smith et al. 2013). Backbone assignments of WT K-Ras are available for aa 1–166 (Vo et al. 2013) and aa 1–180 (Gossert et al. 2011; although a number of residues were undetected in their 2D ^1^H–^15^N HSQC spectrum).

Despite the growing interest in K-Ras G12C as a therapeutic target, no assignments have been reported for this oncogenic mutant in either the GDP- or GTP-bound state. Here we present the ^1^H,^13^C,^15^N backbone NMR assignment of the GDP-bound form of G12C K-Ras (K-Ras^G12C-GDP^), encompassing residues 1–169, at pH 7.0. Under these solution conditions all the backbone amide signals were observed. Chemical shift differences in the 2D ^1^H–^15^N HSQC spectra of the GDP-bound forms of WT K-Ras (Vo et al. 2013) and of G12C mutant K-Ras (this study) are discussed.

## Methods and experiments

### Protein expression and purification

DNA encoding residues 1–169 of K-Ras4B^G12C^ was sub-cloned into a pET28a vector with a N-terminal hexa-histidine (6-His) tag followed by the TEV protease cleavage site. Protein was expressed in *E. coli* BL21(DE3) cells (Novagen), and induced by IPTG at the optical cell density between 0.5 and 0.7. IPTG-induced cells were grown at 30 °C for 16 h. Proteins uniformly labeled with ^15^N or ^13^C/^15^N were prepared by growing *E. coli* cells in minimal media containing ^15^NH_4_Cl (1 g/l) or ^13^C_6_ glucose (2 g/l)/^15^NH_4_Cl (1 g/l) (Cambridge Isotope Laboratories; CIL) supplemented with Bio-Express 1000 (CIL) using the identical protocol used for LB media. Harvested cells were suspended in Buffer A (50 mM Tris, 500 mM NaCl, 10% Glycerol, 1 mM PMSF, 1 mM TCEP, pH 8.0) supplemented with complete protease inhibitor cocktail tablet (Roche) and lysonase (Novagen) (10 μl/l cell culture), agitated 30 min at 4 °C, and sonicated (Branson digital sonifier, S-450D) at 4 °C, 7 × 7 s cycles at 35% amplitude with 1 min on ice between cycles. Supernatant lysate was loaded onto pre-equilibrated (in Buffer A) Ni-NTA column (Qiagen). After 1 h of mixing the lysate, beads were washed 20 column volume (cv) in Buffer B (50 mM Tris, 300 mM NaCl, 10 mM imidazole, 1 mM TCEP, pH 8.0). Protein was eluted in Buffer C (50 mM Tris, 300 mM NaCl, 250 mM imidazole, 1 mM TCEP, pH 8.0). Ni-NTA purified protein was dialyzed in Buffer D (50 mM Tris, 300 mM NaCl, 1 mM TCEP, pH 8.0) and digested with TEV protease at 4 °C overnight. Tagless protein incorporating a Ser residue at the N-terminus (due to cloning exigencies) was recovered by passing through a Ni-NTA column. Purified protein, as detected by Coomassie Blue Staining after SDS-PAGE, was a single band of ~ 19 kDa with apparent purity of > 96%. Protein was further purified by size exclusion chromatography on a Superdex 75 column (GE Healthcare) pre-equilibrated in buffer E (50 mM Tris, 50 mM NaCl, 1 mM MgCl_2_, 1 mM TCEP, pH 8.0). The GDP-bound state of the purified protein was ascertained by HPLC.

### NMR spectroscopy

NMR samples of ^13^C/^15^N- and ^15^N labeled K-Ras^G12C-GDP^ (0.5–0.6 mM) were prepared in a 93% H_2_O/7% D_2_O solvent composition containing 50 mM TRIS-d_11_, 1 mM TCEP-d_16_, 1 mM MgCl_2_, 100 µM 2,2-dimethyl-2-silapentanesulfonic acid (DSS) as internal standard, and 0.05% (w/v) NaN_3_ to avoid any unwanted bacterial growth over time. All NMR experiments were performed at 298 K on a Bruker Avance 800 MHz spectrometer equipped with a 5-mm TCI cryoprobe. NMR data were acquired in the gradient-selected sensitivity-enhanced mode. Backbone ^1^H, ^15^N, ^13^C^α, 13^C^β^, and ^13^CO assignments were carried out using double and triple resonance experiments of 2D ^1^H–^15^N HSQC, 2D ^1^H–^15^N TROSY, HNCACB, HN(CO)CACB, HNCA, HN(CO)CA, CC(CO)NH, and HNCO (Sattler et al. 1999). These NMR data were processed on an Intel PC workstation running Redhat Linux 7.1 using NMRPipe/NMRDraw (Delaglio et al. 1995). The ^1^H, ^13^C, and ^15^N chemical shifts were referenced to the internal standard DSS using IUPAC-IUB recommended protocols (http://www.bmrb.wisc.edu/ref_info/cshift.html). All NMR spectra were visualized and analyzed using CCPNMR analysis (Vranken et al. 2005).

## Assignments and data deposition

**Fig. 1:**
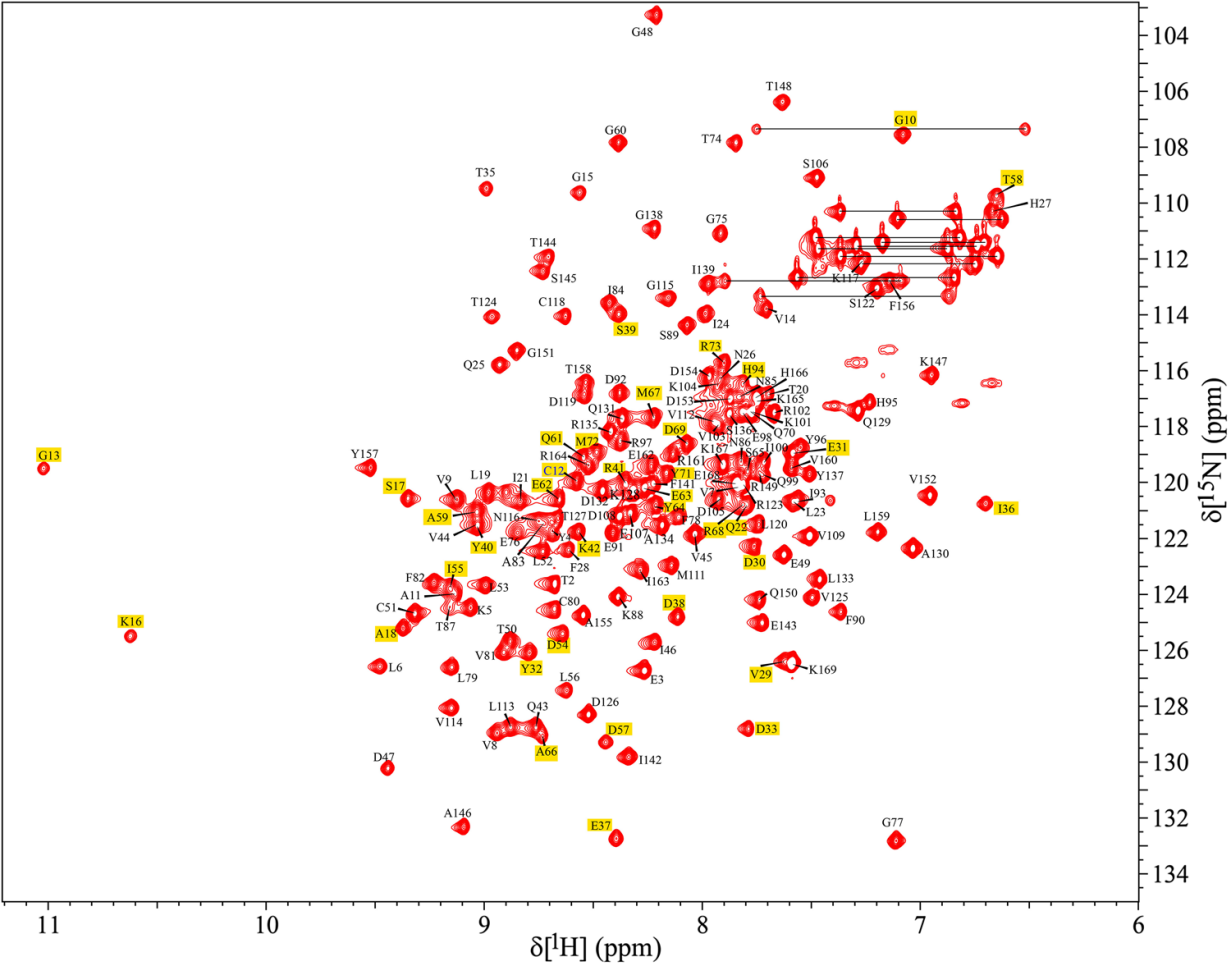
Two-dimensional ^1^H–^15^N HSQC spectrum illustrating the assigned residues from a ~ 0.5–0.6 mM ^13^C/^15^N K-Ras^G12C-GDP^ sample in 50 mM TRIS-d_11_, 1 mM TCEP-d_16_, 1 mM MgCl_2_, 100 µM DSS in 93% H_2_O and 7% D_2_O, pH 7.0 at 298K. Spectrum was recorded on a Bruker Avance 800 MHz spectrometer at 298 K. The assignments shown are annotated using the one letter amino acid code followed by the sequence number of that residue. Unassigned side-chain N–H correlations belong to Asn and Gln (connected by horizontal lines) and Arg. Residues highlighted in yellow are not seen in GTP bound form of K-Ras^G12C-GMPPNP^ (unpublished data)

The assignment of ^1^H^N, 13^C, and ^15^N chemical shifts of human K-Ras^G12C-GDP^ has been deposited into BMRB (http://www.bmrb.wisc.edu/) with accession number 27387. Crosspeaks in the 2D ^1^H–^15^N HSQC spectrum demonstrates that the protein is well-folded in solution under the chosen condition (Fig. 1), and a total of 164 out of 165 non-proline ^1^H–^15^N correlation crosspeaks (99.4%) have been identified and assigned. The amide signal belonging to the first methionine residue could not be visualized presumably due to the exchange of ^1^H^N^ protons with the bulk solvent. The chemical shifts of ^13^C^α, 13^C^β^, and ^13^C′ for all residues (including the Met1 and the 4 proline residues) are assigned using the triple resonance data. Residues Gly13 and Lys16 show the most downfield-shifted ^1^H^N^ chemical shifts, a pattern also seen for GDP-bound WT K-Ras (Vo et al. 2013) and for GDP-bound H-Ras^G12V^. Due to the cysteine substitution at position 12 the ^1^H^N^ chemical shift of Gly13 is further downfield shifted to its WT counterpart (Vo et al. 2013). Although the chemical shift dispersion of residue crosspeaks in the 2D ^1^H–^15^N HSQC spectra of WT and G12C K-Ras is apparently similar, the notable differences in the chemical shifts are observed for select residues. The most significant is the ^15^N chemical shift of the residue 12 (120 ppm in the G12C vs. 106.7 ppm in the WT) (Vo et al. 2013). Other residues which experience notable mutation-induced changes in the ^1^H–^15^N correlation chemical shift are Gly13, His27, Val44, Gly48, Ala66, Gly77, Thr87, and His94. The differences in peak positions of Gly48 and Gly77 could be due to their aliasing in ^15^N dimension. C-terminal residues Arg164, Lys165, and His167 also show significant changes in their peak positions in 2D ^1^H–^15^N HSQC spectrum of G12C to that of WT counterpart (Vo et al. 2013).

The study presented herein has two experimental differences compared to the study of WT protein (Vo et al. 2013). First, the current study (K-Ras^G12C^; 1–169) uses a construct which is 3 residues longer at C-terminus than WT K-Ras (1–166). Second, while the NMR assignment reported for WT K-Ras used a perdeuterated sample, non-deuterated NMR samples were used in this study to yield high-resolution NMR data and completion of unambiguous NMR assignments.

### Extent of assignment

**Fig. 2:**
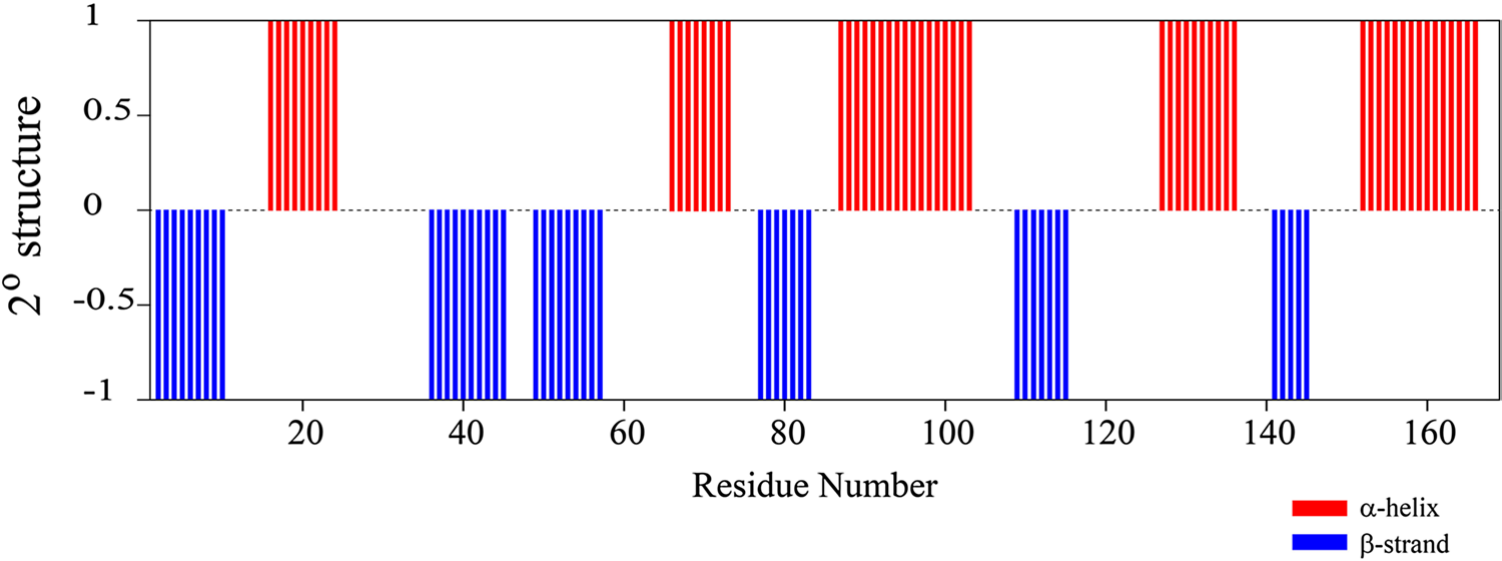
The consensus chemical shift index (^1^H^α, 13^C^α, 13^C^β, 13^C^′, 15^N, ^1^H^N^) versus residue number plot of K-Ras^G12C-GDP^ showing β-strands (represented by bars with CSI value of − 1) and α-helices (represented by bars with CSI value of 1)

In total, assignments were made for 95.5% of the backbone resonances, including ^1^H^N, 15^N, ^13^C^α^, and ^13^C^β^. 99.4% of non-proline backbone ^1^H–^15^N correlation crosspeaks were assigned (^1^H–^15^N correlation crosspeak for Met1 is not seen in 2D ^1^H–^15^N TROSY spectrum). Side-chain ^13^C assignments were achieved for 224 out of possible 269 aliphatic carbon resonances (including 95.9% of ^13^C^β^ assignments). 100% of 13C^α^, 98.8% of 13C^β^ (crosspeaks not seen for Ser17 and Thr158), and 96.5% of ^13^CO resonances were assigned unambiguously. A chemical shift index (CSI) v 3.0 (Hafsa et al. 2015; Berjanskii and Wishart 2005) consensus for the ^13^C^α, 13^C^β^, and ^13^C′ atoms suggest that the K-RAS^G12C-GDP^ conformation comprises a mixed distribution of α helix and β sheet secondary structures (Fig. 2). A total of 62% residues are engaged in constituting 11 canonical secondary structural elements that are arranged in the order of β_1_–α_1_–β_2_–β_3_–α_2_–β_4_–α_3_–β_5_–α_4_–β_6_–α_5_, and match well with the secondary structure order observed in the GDP-bound crystal structure of K-Ras G12C (Hunter et al. 2014). The lengths of the secondary structure segments deduced from CSI are in good agreement with those noted in the three-dimensional structural fold generated using CS-Rosetta (Shen et al. 2008).

In conclusion, we present the backbone NMR assignment of GDP-bound K-Ras harboring an oncogenic cysteine mutation at site 12. Efforts are underway to collect additional NMR data to complete all the side-chain resonances of KRas^G12C-GDP^.
